# A Suggestion or Aid to the Eradication of Tuberculosis by a Practical and Profitable Method

**Published:** 1895-08

**Authors:** A. S. Heath


### A SUGGESTION OR AID TO THE ERADICATION OF TUBERCULOSIS BY A PRACTICAL AND PROFITABLE METHOD.

BY. A. S. HEATH, M.D., V.S.

NEITHER the wholesale slaughter of tuberculous herds nor the wholesale defamation of the character of the veterinary profession can ever result in public good. The disease is a menace to public health and life and a fearful waste of property, both private and public. The safe and judicious accumulation of private property is a public benefaction, and any profession that protects health, life, and property is a conservator of public health and wealth. Both branches of the profession, the human and the comparative, are preservers and protectors of public and private health and wealth. The human physician prevents and cures disease in man, while the veterinarian performs the same noble office in animals, that man shall not suffer from the diseases of animals; so the hygiene of both branches of our noble profession is related as well as co-operative. But now superadded to the function of the veterinarian is the conservation of private property. Every animal has a certain property valuation. It may be one dollar or one thousand dollars. And when the veterinarian saves the health, product, and life of these animals, he saves not only property, but he also protects the health and life of the public. Both branches of our grand profession are eminently humane, for both are sanitarian in their highest functions.

It is also true that the health and lives of men and animals have a relative property value, and for a monetary consideration the great insurance corporations insure owners and interested parties against loss. These worthy institutions render a vastly greater service than is generally accredited to them. A man whose life is insured feels less anxiety for his family and friends, and he lives longer. So he who insures his animals feels relief and assurance against sudden and total loss. There is a question whether the loss of stock by contagious diseases should be borne wholly by the owner, or in part by the State. The loss is both a private and a public loss. But how much is due to private or public negligence demands honest consideration. Though these considerations may not properly belong to the present subject before us, yet they are worthy of contemplation.

The owner of cattle suspects from some unusual conditions in his herd that tuberculosis may be present. His interest demands prompt action, and his intelligence and judgment dictate the calling of a veterinarian. When put in charge, common sense says submit to professional advice and judgment. The veterinarian diagnosticates the case or cases by every means at his command. He knows that on the use of tuberculin his main reliance rests, and that if used in cases where there is no infection no harm results. In cases of doubt or uncertainty the veterinarian calls counsel. In cases of tuberculosis the post-mortem confirms the diagnosis. It will be well to invite the family physician to be present to aid in the post-mortem. This is professional etiquette, as well as good judgment, for it establishes friendly relations which will and should prove valuable to all concerned.

All suspected animals after tuberculization, if not slaughtered, should be separated from the non-suspected. And the stables of these should be cleansed and disinfected, and all proper hygienic measures employed. The veterinarian has not yet completed his work. He must see that the herd is free from the disease, and that no untested animals should be added to it. And if the herd is a breeding one, none but sound healthy animals should be bred together. Further still, he should advise the owner to breed from such males as are known to belong to a breed that is not susceptible to tuberculosis. This presupposes that the veterinarian is versed in such matters. The owners are generally supposed to know what they want and what breed they prefer. If a dairy herd, the males should possess the blood of the characteristic excellence for the dairy; or if a beef herd, the beef-blood. But sound health, vigor, and constitution must be demanded for any and all products.

Now probably neither the owner nor the veterinarian knows anything about the superior combined excellences of the Norman for both milk and meat; nor that this Norman has no tendency to tuberculosis; nor that it is one of the most healthy, hardy, vigorous, gentle, large, and useful dairy-and-beef-combined breeds of the world. And that breeding up grades by crossing males of this breed on both healthy dairy and beef cows the size, quality, stamina, and freedom from tuberculosis must be insured, as well as the combined excellences for milk and beef secured. Both of these excellences, and the resisting power to disease, have been imparted to many herds of Europe in proportion to the amount of the blood-infusion received from the noble Cotentin of Normandy. Similar occurrences took place in the various breeds of draft horses, from the old black Flemish draft horse. This blood permeated all the heavy draft horses of the world.

The Norman is a large animal. The cows attain at maturity 1200 to 1600, or more; the oxen when fat for market, from 2200 to even 4000 pounds. The head is only, like the bone, relatively large, the body long and deep, the hips broad, the legs short and stout, the skin thick but mellow, the coat red roan, spotted about the head, shoulders, and streaked with brindle; some have white faces and some are cream-fawn; the udders are large, deep, and broad, but placed high up, though yielding a large flow of milk—twenty, twenty-five, thirty, and even forty quarts from three daily milkings; and the Isignay butter brings at the Halles Centrales at auction in Paris the highest price the year through. The veal from the Normandy stock is the largest and best in the Paris and other markets.

It is this substance and stamina that resist tuberculosis, and is the hope for the eradication of the disease in our dairy herds. Grades of this blood will add to the Jersey and Guernsey, whose progenitors the Normandy is, at least one-third more size, give tone and constitution, increased products of beef, as well as dairy products.

It is this increase of health and dairy and beef products that will add value to all our herds. The increase of milk to the beef herds will tell promptly on the calves, veals, cows, and bullocks in size and quality of meat.

Registered stock have added in crossing on the Norman the Norman register, and with the Jersey and Guernsey the renewal of the original blood with the original sound health. Enough, perhaps, for the present.

